# Integrative analysis based on survival associated co-expression gene modules for predicting Neuroblastoma patients’ survival time

**DOI:** 10.1186/s13062-018-0229-2

**Published:** 2019-02-13

**Authors:** Yatong Han, Xiufen Ye, Jun Cheng, Siyuan Zhang, Weixing Feng, Zhi Han, Jie Zhang, Kun Huang

**Affiliations:** 1 Department of Automation, Harbin Engineering University, Harbin, China; 2 Department of Neurosurgery, Stanford University, California, USA; 3 Department of Medicine, Indiana University School of Medicine, Indianapolis, USA; 4 Department of Medical and Molecular Genetics, Indiana University School of Medicine, Indianapolis, USA; 5 School of Biomedical Engineering, Shenzhen University, Shenzhen, China; 6 Regenstrief Institute, Indianapolis, USA

**Keywords:** Neuroblastoma survival time predict, Gene co-expression network, Integrative cluster

## Abstract

**Background:**

More than 90% of neuroblastoma patients are cured in the low-risk group while only less than 50% for those with high-risk disease can be cured. Since the high-risk patients still have poor outcomes, we need more accurate stratification to establish an individualized precise treatment plan for the patients to improve the long-term survival rate.

**Results:**

We focus on extracting features and providing a workflow to improve survival prediction for neuroblastoma patients. With a workflow for gene co-expression network (GCN) mining in microarray and RNA-Seq datasets, we extracted molecular features from each co-expressed module and summarized them into eigengenes. Then we adopted the lasso-regularized Cox proportional hazards model to select the most informative eigengene features regarding association to the risk of metastasis. Nine eigengenes were selected which show strong association with patient survival prognosis. All of the nine corresponding gene modules also have highly enriched biological functions or cytoband locations. Three of them are unique modules to RNA-Seq data, which complement the modules from microarray data in terms of survival prognosis. We then merged all eigengenes from these unique modules and used an integrative method called Similarity Network Fusion to test the prognostic power of these eigengenes for prognosis. The prognostic accuracies are significantly improved as compared to using all eigengenes, and a subgroup of patients with very poor survival rate was identified.

**Conclusions:**

We first compared GCNs mined from microarray and RNA-seq data. We discovered that each data modality yields unique GCNs, which are enriched with clear biological functions. Then we do module unique analysis and use lasso-cox model to select survival-associated eigengenes. Integration of unique and survival-associated eigengenes from both data types provides complementary information that leads to more accurate survival prognosis.

**Reviewers:**

Reviewed by Susmita Datta, Marco Chierici and Dimitar Vassilev.

**Electronic supplementary material:**

The online version of this article (10.1186/s13062-018-0229-2) contains supplementary material, which is available to authorized users.

## Background

**Fig. 1:**
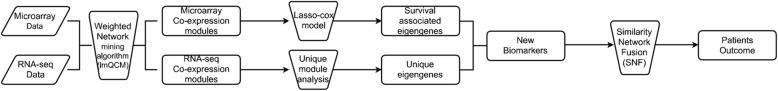
Graphical representation of the Integration workflow

Neuroblastoma (NB) is one of the most common cancers in children. The patients of high-risk (HR) subtype usually have the poorer prognosis [1]. Better survival prediction for these HR patients will help doctors adjust their treatment plans, thus improve the patient’s chances of survival. With abundant high-throughput transcriptomic data [2–4], a better prognosis method may benefit from an integrative approach which extracts highly correlated molecular features and identifies them as potential biomarkers for patient survival prognosis [5]. However, there are two major challenges for the integrative approach: (1) the relatively small number of samples compared to a large number of measurements; and (2) complementary nature of the information provided by different types of data [6, 7]. In this paper, we provide an effective workflow to tackle these problems, the workflow is shown in Fig. 1. For complementary nature in NB transcriptomic data, a study has compared RNA-Seq and Agilent microarray gene expression profiles for clinical endpoint prediction of 498 pediatric patients and found the two technology platforms do not significantly affect performances of the models [8]. However, instead of examining data for the large number of genes, which contain noise and poses a problem on the statistical power of prognosis, we reduce the data dimensionality by mining gene co-expression network (GCN) first. Specifically, we identified densely connected GCN modules, then summarized each module into an “eigengene” using the protocol described in [9, 10]. To distinguish this study from another study we did on NB, which was focus on efficiently integration of the transcriptomic data and clinical data using consensus clustering, in this paper we probed into details for these eigengenes and their biological functions, and identified GCN modules that can be used as potential biomarkers to improve accuracy for NB patient survival prognosis. Therefore, after the eigengene construction and analysis, we built a lasso-regularized Cox proportional hazards (lasso-Cox) model to compute the risk index for each patient in the HR group with all the eigengenes to identify the ones significantly contributing to the prediction. Finally, we applied an integrative method called Similarity Network Fusion (SNF) [11] to merge these eigengenes and test the power of their prognostic power as potential biomarkers.

**Table 1:** *P*-value of Correlation Index of genes with 10 unique RNA-seq modules in RNA-seq data

	R7	R9	R13	R15	R17
*P*-value	0.001	0.001	0.001	0.001	0.001
	R20	R21	R22	R23	R24
*P*-value	0.001	0.001	0.001	0.001	0.001

## Materials and methods

### Dataset and preprocessing

The data used in this study is obtained from the Neuroblastoma Data Integration Challenge of CAMDA 2017. It contains tumor samples of 498 neuroblastoma patients from seven countries: Belgium (*n* = 1), Germany (*n* = 420), Israel (*n* = 11), Italy (*n* = 5), Spain (*n* = 14), United Kingdom (n = 5), and United States (n = 42). The patients’ age at diagnosis varied from 0 to 295.5 months (median age, 14.6 months).

Transcriptome datasets from both microarray (Agilent 44 K oligomicroarray) and RNA-seq are obtained for the 498 patients with known clinical endpoints. The RNA-seq includes 60,788 transcripts and Agilent microarray data for 45,198 probesets, both from 498 primary neuroblastomas. Tumor stage was classified according to the International Neuroblastoma Staging System (INSS): stage 1 (*n* = 121), stage 2 (*n* = 78), stage 3 (*n* = 63), stage 4 (*n* = 183), and stage 4S (*n* = 53). 176 patients are labeled as high-risk, which are the patients with stage 4 disease more than 18 months at diagnosis and patients of any age and stage with MYCN-amplified tumors [1]. We identified 9583 unique genes whose expression profiles are present in both RNA-seq and microarray datasets with matched gene symbols for further analysis and data integration.

### Gene co-expression analysis and eigengene summarization

While our first goal is to extract these gene data feature before integration, the large gene number poses a challenge on the statistical power. Therefore, instead of focusing on individual genes, we first carry out gene co-expression network analysis (GCNA) to cluster genes into co-expressed modules and summarize each module into an “eigengene”. This approach not only substantially improves statistical power but also allows us to focus more on important biological processes or genetic variations associated with the co-expressed gene modules, making the results more interpretable. We applied our recently developed weighted network mining algorithm local maximum Quasi-Clique Merging (lmQCM) for GCN mining [12]. Unlike the widely used WGCNA package that uses hierarchical clustering and does not allow overlaps between clusters [13, 14], lmQCM is a greedy approach and allows genes to be shared among multiple clusters, agreeing with the fact genes often participate in multiple biological processes. Also, it has been shown to be able to find smaller co-expressed gene clusters that are often associated with structural mutations such as copy number variations in cancers. The adjacency (weight) matrix was constructed using Spearman Correlation Coefficient (SCC) for every pair of gene studied, as SCC can accommodate the large non-linear range of RNA-Seq data better than Pearson Correlation Coefficient. Four parameters in lmQCM algorithm need initialization, they are *λ*, *α*, *t*, and*β*. Among them, *λ* is the most important one. It determines the initiation of a new cluster by setting the weight threshold for the first edge of the cluster as a sub-module. In our GCN analysis, we transform the absolute values of the SCC between expression profiles of genes into weights using a normalization procedure adopted from spectral clustering [14], which has been shown to be effective in previous studies. Based on previous work [15, 16], we chose *λ*=0.80, *t* = 1, *α* = 1, and *β*= 0.4, which yielded 38 co-expressed gene clusters from microarray and 24 co-expressed gene clusters from RNA-seq with balanced sizes and clear biological interpretations.

### Lasso-regularized cox proportional hazards model

After using lmQCM reduced data dimension, we want to find more important survival-associated modules as features of subsequent integration algorithms. Thus, we built a lasso-regularized Cox proportional hazards (lasso-Cox) model to compute the risk index of each patient, using the eigengenes generated from GCN [18]. Lasso penalty (i.e. L1 penalty) generates sparsity and outputs an informative subset of features [19]. To help select the parameters, we used a two-level cross validation (CV) strategy - first leave-one-out CV then 10-fold CV to select the optimal regularization parameter. Regularized Cox proportional hazards model was built on the training set using the selected parameter to compute the risk indices of all patients. After that, patients were split into low-risk and high-risk groups according to the median of risk indices of the training examples. At last, we tested if these two groups have distinct survival outcome using Kaplan-Meier estimator and log-rank test, where p less than 0.05 was considered significant. Since our initial goal is to screen for all possible survival-associated features, we did not apply multiple test compensation control such as FDR. The lasso-Cox model was trained on the selected survival-associated features. Cox proportional hazards regression model was applied, and 95% confidence intervals were computed to determine the prognostic values of our lasso-Cox risk indices and clinical stage.

With the lasso-regularized Cox proportional hazards model, we can obtain eigengenes that are strongly associated with survival times. However, if only consider selected features of one dataset were used to predict the endpoint of patients, it will result in the lack of information as this lasso Cox model is based on the median of risk indices of the training examples. To obtain a more reasonable classification result, a more effective way might be to make full use of all the information, but we know that in essence microarray and RNA-Seq data are the same, it calls for extra caution to incorporate these datasets.

We use two steps to address this problem: First, based on the unique module analysis, we can identify the unique modules in survival-associated features (co-expression modules) selected by Lasso-cox model of each gene dataset. Secondly, respectively building patients similarity network based on about unique modules in each gene dataset, then integrate these two networks. The Similarity Network Fusion(SNF) is a state-of-the art network integrative method and is adopted here.

### Unique module analysis

We used Jaccard index less than 0.05 and Fisher exact test *p*-value greater than 0.05 as the metrics to determine the uniqueness of co-expression modules between the Microarray and RNA-Seq data (Additional file 1: Table S1).

**Table 2:** P-value of Correlation Index of genes with 17 unique Microarray modules in Microarray data

	M3	M4	M5	M8	M9	M11	M13
*P*-value	0.001	0.001	0.001	0.001	0.001	0.001	0.001
	M19	M20	M21	M22	M28	M30	M31
*P*-value	0.001	0.001	0.001	0.001	0.001	0.001	0.001
	M32	M34	M38				
*P*-value	0.001	0.001	0.001				

### Evaluation of modules

In order to further evaluate the correlative relationship of genes within each module, we also introduced the term Correlation Index using SCC matrix [17]. Correlation Index (C) of a module with K genes is formulated as:

1 $$ C=\frac{{\left\Vert W-{I}_{K\times K}\right\Vert}_F^2}{K^2} $$

*P*-value is also computed for each C value by randomly selecting K genes for 1000 times that sampling was done within the given module, and calculating Correlation Index (C*) each time

2 $$ p=\frac{\#\left({C}^{\ast }>C\right)}{1000} $$

### Similarity network fusion (SNF)

SNF [11] construct similarity weight matrix (patients network) of the sample for each available data type and then fusing these into one network that represents the full spectrum of underlying data. There are three parameters in SNF: K is the number of neighbors, α is a hyperparameter, *t* is the number of Iterations. We found that by setting the three parameters to 30, 0.8, and 20, respectively, it can obtain the best classification result.

The key step of SNF is to iteratively update similarity weight matrix corresponding to each of the data types as follows:

3 $$ {\tilde{W}}_{t+1}^{(1)}={S}^{(1)}\times {W}_t^{(2)}\times {S}^{(1)T} $$

4 $$ {\tilde{W}}_{t+1}^{(2)}={S}^{(2)}\times {W_t}^{(1)}\times {S}^{(2)T} $$

Where ${W}^{\left(\overset{\frown }{m}\right)}$ is defined as:

5 $$ {\tilde{W}}^{(m)}=\left\{\begin{array}{l}\frac{W_{i,j}^{(m)}}{2\sum \limits_{k\ne i}{W}_{i,k}^{(m)}}\\ {}\frac{1}{2}\end{array}\right.{\displaystyle \begin{array}{l} if\kern0.5em i\ne j\\ {} if\kern0.5em i=j\end{array}} $$

Let *D(i)* represent a set of *x*_*i*_’s neighbors including *x*_*i*_ in *G*. Given a graph, *G*, we use K nearest neighbors (KNN) to measure local affinity. So *S*^*(m)*^ is defined as:

6 $$ {S}_{i,j}^{(m)}=\left\{\begin{array}{l}\frac{W_{i,j}^{(m)}}{2\sum \limits_{k\in {N}_i}{W}_{i,k}^{(m)}}\\ {}0\end{array}\right.{\displaystyle \begin{array}{l} if\kern0.5em i\ne j\\ {} if\begin{array}{cc}& otherwise\end{array}\end{array}} $$

That ${W}^{\left(\overset{\frown }{m}\right)}$ carries the full information about the similarity of each patient to all other patients whereas S^(m)^ only encodes the similarity to the K most similar patients for each patient. This procedure updates the weight matrices each time generating two parallel interchanging diffusion processes. After t steps, the overall weight matrix is computed.

7 $$ {W}^{\ast}\left(i,j\right)=\frac{{\tilde{W}}_t^{(1)}\left(i,j\right)+{\tilde{W}}_t^{(2)}\left(i,j\right)}{2} $$

### Enrichment analysis of the gene set

The online gene list enrichment tool ToppGene (http://toppgene.cchmc.org) developed by Cincinnati Children’s Hospital Medical Center [20] was used for all of the module functional enrichment analysis. ToppGene not only carries out enrichment analysis on standard Gene Ontology, it also generates enrichment results from more than 20 different sources including pathway databases, human and mouse phenotypes, NCBI PubMed, transcription factor binding sites, and drug information.

## Results

### Co-expression modules compared between microarray gene expression and RNA-seq

**Fig. 2:**
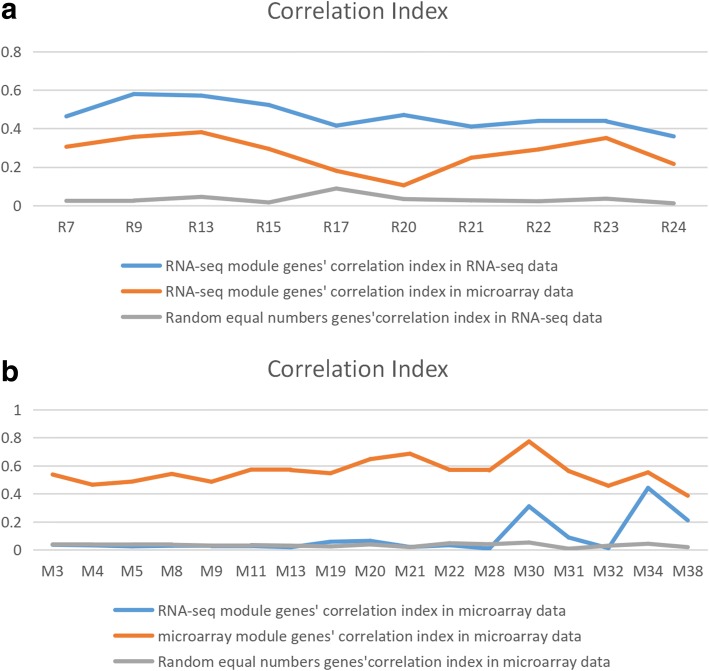
log(Correlation index) in different data. **a.** Correlation index with each unique microarray module genes in microarray, RNA-seq data, and equal number random genes in microarray data. **b.** Correlation index with each unique RNA-seq module genes in RNA-seq data, microarray,and equal number random genes in RNA-seq data

Previous studies compared RNA-Seq and Agilent microarray gene expression profiles for clinical endpoint prediction of 498 children patients. Evaluation of factors potentially affecting model performances reveals that prediction accuracies are most strongly influenced by the nature of the clinical endpoint, whereas technological platforms (RNA-Seq vs. microarrays), RNA-Seq data analysis pipelines, and feature levels (gene vs. transcript vs. exon-junction level) do not significantly affect performances of the models [2]. But these studies did not focus on the comparison of co-expression network structures and the GCN modules in these two kinds of data. After applying lmQCM, 38 co-expression modules from microarray and 24 from RNA-seq were identified. In order to determine if data modality affects the correlation as well as modules identified, a comparison was performed between each pair of modules from microarray and RNA-seq. Among them, 17 GCN modules from microarray and 10 from RNA-seq are unique to its own data type (Additional file 1: Table S1), and several of them are enriched with different biological processes, molecular functions, or specific pathways related to cancer physiology or neurological functions (Additional file 2: Table S2). We also tested the correlation of the genes in testing sets use concordance index (a metric we developed previously to test the correlation of genes in a co-expressed module) to test their stability. The result shown in Supplement (Additional file 3: Figures S1 and Additional file 4: S2). By computing the correlation indices of these unique modules (Tables 1, 2, 3 and 4), we discovered that most of the unique GCN modules from the RNA-seq data are not highly correlated in microarray data (Fig. 2(a)), whereas the unique GCNs in the microarray data are often correlated in RNA-seq data (Fig. 2(b)).

**Table 3:** P-value of Correlation Index of genes with 10 unique RNA-seq modules inMicroarray data

	R7	R9	R13	R15	R17
*P*-value	0.001	0.001	0.001	0.001	0.001
	R20	R21	R22	R23	R24
*P*-value	0.001	0.001	0.001	0.001	0.001

**Table 4:** P-value of Correlation Index of genes with 17 unique Microarray modules in. RNA-seq data

	M3	M4	M5	M8	M9	M11	M13
*P*-value	0.981	0.992	0.999	0.995	0.999	0.999	0.999
	M19	M20	M21	M22	M28	M30	M31
*P*-value	0.199	0.125	0.943	0.662	0.993	0.001	0.061
	M32	M34	M38				
*P*-value	0.953	0.001	0.001

### Survival-associated gene modules

Nine survival-associated eigengenes were selected by using (lasso-Cox) model. Among them, five are the survival-associated eigengenes from microarray data (M2, M7, M10, M36, and M37), and four from RNA-seq (R2, R7, R17, R21). Especially, R7, R17, R21 are from RNA-seq only modules, these modules are not present in Microarray data. Most of the nine modules are highly enriched with biological functions: M2 (127 genes) and R2 (268 genes) are highly enriched with cell cycle genes (containing 39 and 64 cell cycle genes respectively and Bonferroni-corrected-*p*-values being 1.05e-70 and 3.88e-78). M10 and M37 are highly enriched with immune response genes, M7 is highly enriched with extracellular matrix organization genes (*p*-value 3.01e-12). All of these agree with the previous pan-cancer study that the top three most common GCN in cancer are cell cycle, immune response and extracellular matrix organization genes [21]. M36 contains no enriched molecular function or biological process, but five of the genes are co-localized on the same cytoband, which indicates a potential structural variant in NB patients. R17 and R21 are enriched with RNA polymerase II transcription regulatory genes.

### Prognostic prediction based on integrative method

**Fig. 3:**
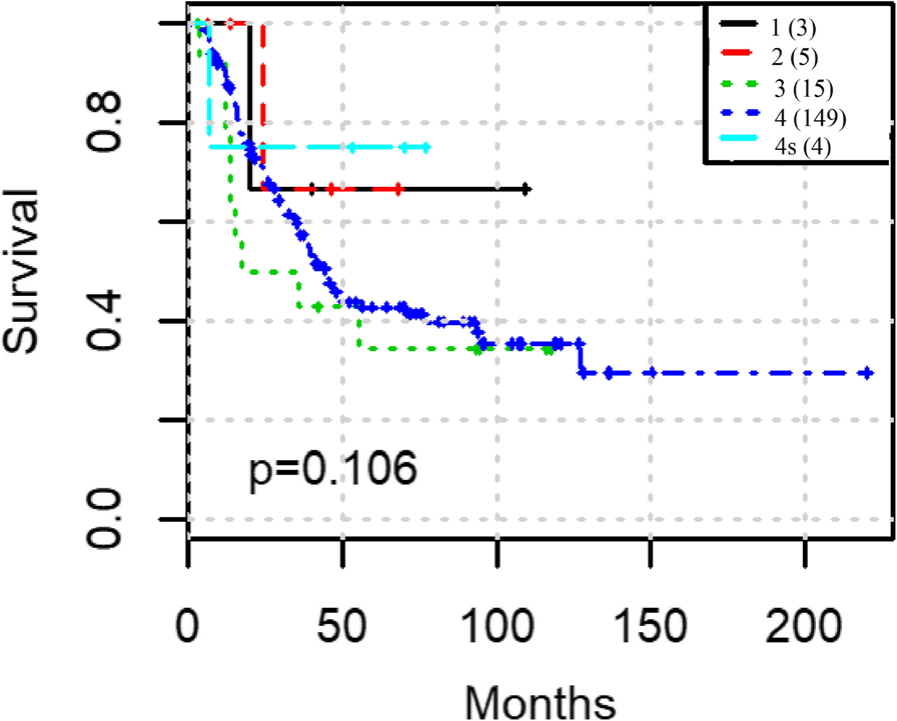
Spectral clustering predict the survival outcomes with different features: (**a**) all 38 eigengenes from microarray data; (**b**) 5 survival associated eigengenes from microarray data; (**c**) all 24 eigengenes from RNA-seq data; (**d**) 4 survival associated eigengenes from RNA-seq data

**Fig. 4:**
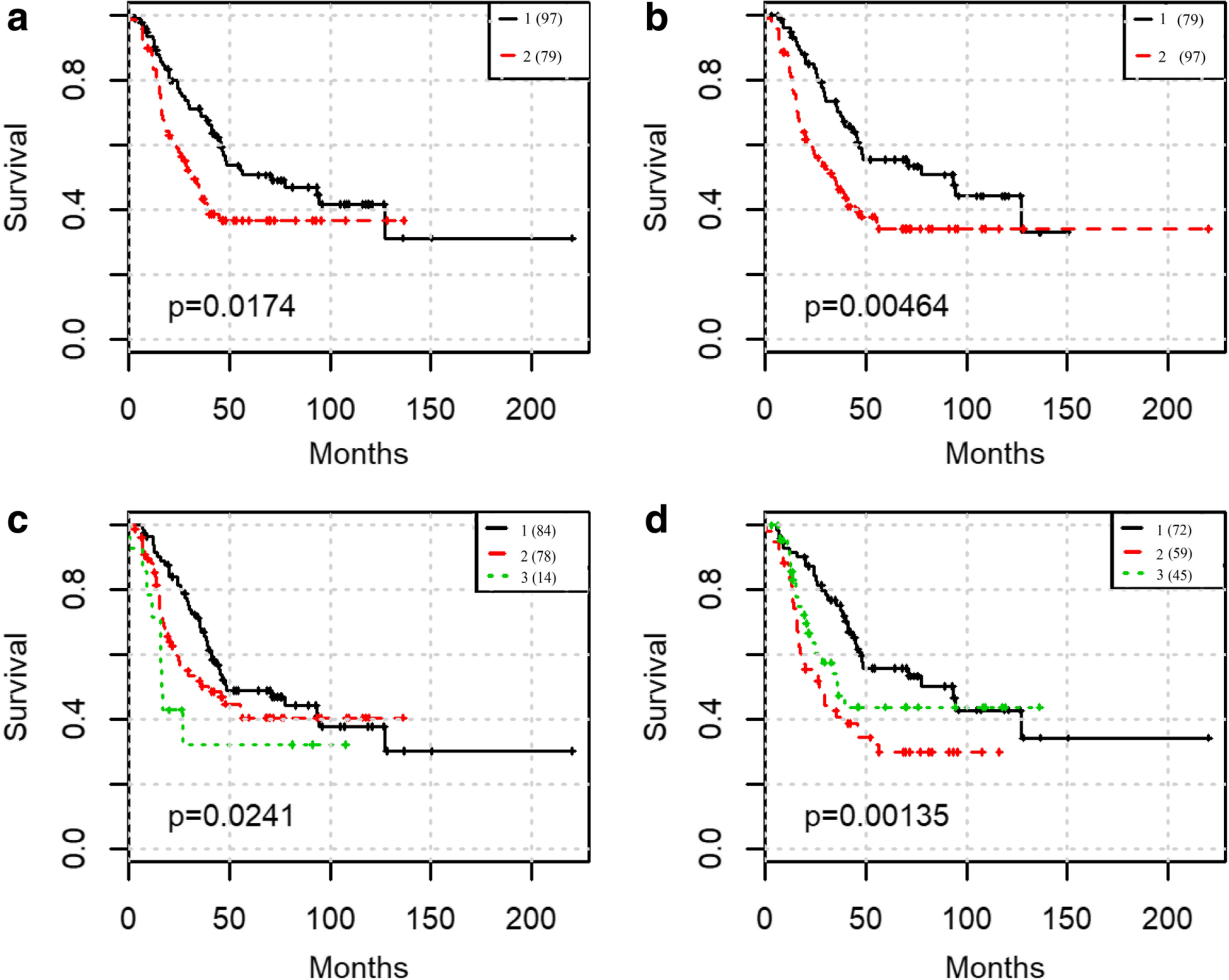
SNF based on 8 unique survival associated co-expression gene modules

**Fig. 5:**
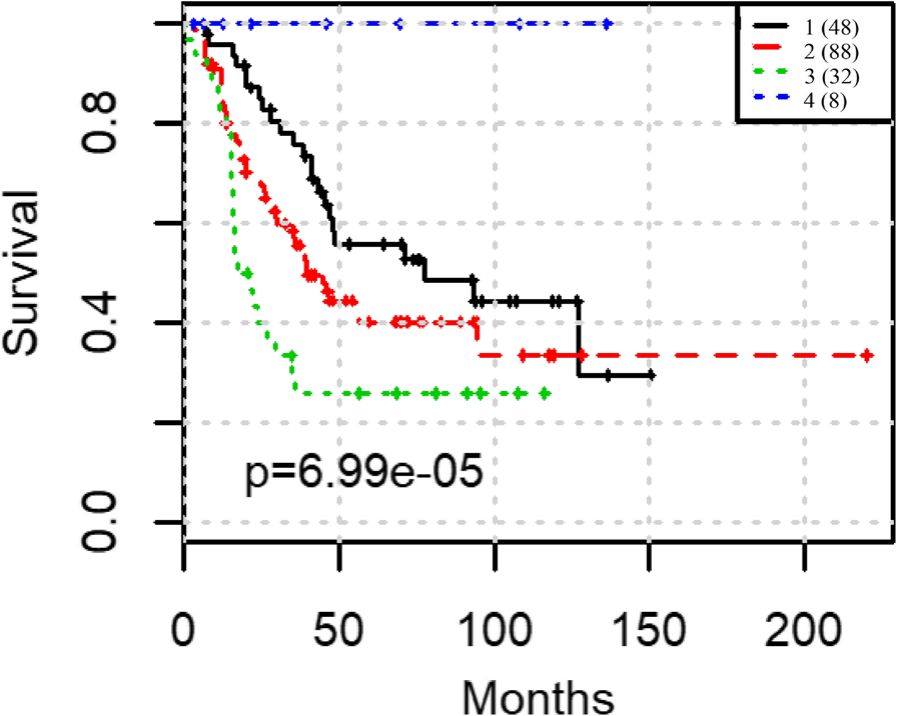
Clinical stage predict the survival outcomes

To test prediction power of our integration workflow, this was carried out in two steps: First, we tested GCNs for prognosis from microarray and RNA-Seq separately and compared the prognosis results between above selected eigengenes with all of the eigengenes in one data type. We used spectral clustering to classify the NB patients first by the 5 selected eigengenes and all 38 eigengenes from microarray, then by the 4 selected eigengenes and all 24 eigengenes from RNA-seq respectively. The results show that the nine selected eigengenes can effectively separate the patients into groups with significant difference in survival times: in microarray data, the *p*-value is reduced from 0.0147 to 0.00464 (Fig. 3(a) and Fig. 3(b)) while in RNA-seq data, the p-value is reduced from 0.0241 to 0.00135 (Fig. 3(c) and Fig. 3(d)). Secondly, we applied SNF approach to integrating five microarray eigengenes with three RNA-Seq eigengenes which were shown to be highly correlated to survival by Lasso-Cox model and unique by the above analysis. The log-rank test p-value is reduced to 6.99e-5 (Fig. 4). The prognosis is also better than using clinical staging (p-value 0.106 Fig. 5). More importantly, the prognosis using the eight eigengenes are able to stratify the high-risk patients further. One additional subgroup of patients with extremely poor survival was identified. The survival rate of the worst group is less than 30% within the first 50 months (Fig. 4).

## Conclusion

In this study, we first compared GCNs mined from microarray and RNA-seq data. We discovered that each data modality yields unique GCNs, which are enriched with clear biological functions. By multivariate lasso-Cox regression analysis, we identified nine survival-associated eigengenes features from microarray data (five eigengenes) and RNA-seq data (four eigengenes) that eight of them is unique. To test the power of the combination of these eight unique eigengenes as prognostic biomarkers, we use spectral clustering as well as SNF for survival prognosis, these eight eigengenes significantly improved the survival prognosis by several magnitudes in terms of log-rank test p-value, as compared to results obtained using all of the modules, modules from one data type, or the clinical stage information. These results suggest instead of focusing on individual genes, using gene co-expression network analysis (GCNA) to cluster genes into co-expressed modules and summarize each module into an “eigengene” is a better way to deal with large number gene data. Module unique analysis and lasso-cox model will further help us choose unique survival-associated eigengenes. Integration of unique and survival-associated eigengenes of both data types provides more complementary information will help achieve a more accurate survival prognosis. Also, we identified one subgroup of patients with very poor survival among high-risk patients. Currently, the underlying reasons for the differences between the GCN structures of the two data modalities are still being investigated.

## Reviewer comments

### Reviewer’s report 1: Susmita Datta

The goal of this paper is to find eigengenes that can serve as potential biomarkers for improving the prognosis of high-risk patients and to give a biological description of these eigengenes. Overall, the authors’ methods and approach are valid (but see major recommendation 1), and their results are promising.

In the methods section, it isn’t clear whether the lmQCM algorithm for determining modules and corresponding eigengenes was applied to the entire dataset or only to the training data. If the former, then the cross validation performed later to assess the performance of the lasso-cox model might be biased. The concern is that, even though the lmQCM is unsupervised (i.e. the survival times aren’t used), if the eigengenes are not stable then using the whole dataset to construct them may lead to underestimation of the error rate during CV (because we are selecting features favorable to both the train and test data). It would be good to check that similar eigengenes are obtained from just the training data alone.

Author’s response: *As correctly pointed out by reviewer, lmQCM does not use any information about the survival and thus it is an unsupervised method. As the reviewer suggested that the eigengenes stability is very important. We therefore tested the correlation of the genes in testing sets use concordance index (a metric we developed previously to test the correlation of genes in a co-expressed module) to test their stability. The result shown in* Additional file 3*: Figure S1 and* Additional file 4*: Figure S2 below. The co-expression modules were first detected from training set, and then the concordance indices were calculated for each gene module in the testing set. The observation is that the concordance indices are stable between the training and testing sets for all the modules and are significantly higher than randomly selected gene sets, which demonstrated the stability of the modules and our approach.*

The primary tool for assessing the prognostic ability of the eigengenes is through Kaplan-Meier (KM) curves and the log-rank test. The KM curve using INSS stage (1, 2, 3, 4, and 4 s) is used as a baseline, however this is not adequate. The stratification of patients into risk groups in practice takes other clinical into variables. For example, MYCN amplification is well known to be highly predictive of high-risk patients. A fair evaluation of the authors’ method would be to use the KM curve constructed using the (clinically evaluated) high-risk indicator that is already provided for each patient. Alternatively, since high-risk patients are of primary interest, the authors can subset on these patients and see whether their method can significantly sub-classify those patients. As it stands, it is not clear if the eigengenes provide any prognostic value beyond that provided by clinical variables currently in use.

Author’s response: *The patients of focus are already labeled as high-risk, which are the patients with stage 4 disease more than 18 months at diagnosis and patients of any age and stage with MYCN-amplified tumors. The MYCN cannot make more contribution for classification of the high-risk patients. But our workflow can give a better classification than use the clinical stage with these patients.*

This study uses overall survival as the outcome, but how does this approach perform for predicting event-free survival? Are there eigengenes that are associated with this outcome as well? And if so, are they different from the ones associated to overall survival.

Author’s response: *We thank the reviewer for this important point. In this paper with the selection of data we focus on overall survival, the event-free survival for events such as relapse and metastasis will require more comprehensive set of data beyond the scope of this paper even though but our methods will be applicable on these data.*

Since copy number variation (CNV) data is available for these patients, and the authors suggest (page 3 line 8) that lmQCM can find modules that are association with structural mutations (like CNV). The CNV data provides an opportunity to verify that claim. It was also mentioned (page 5 line 32) that some M36 genes are “co-localized on the same cytoband, which indicates a potential structural variant in NB patients.” the CNV data can be used to investigate this.

Author’s response: *We totally agree and the integration/comparison with CNV data is part of our ongoing work.*

Page 3, eq. (1): Is this using the Frobenius norm? The norm used is not stated.

Author’s response: *Yes, we clarified this in the revision.*

Page 3, line 46: Computing *p*-values is done by “randomly selecting K genes for 1000 times”. Is this sampling done within the given module or among all genes? If the latter, is it sampling with replacement.

Author’s response: *This sampling is performed within the given module. We provide a more detailed description in the paper.*

Page 4, line 27: “We found that by setting and o be 30, 0.8, 20 respectively, …” contains typos. Consider “We found that by setting the three parameters to 30, 0.8, and 20, respectively”.

Author’s response: *We revised the description.*

8. Page 5–6: The figure references do not match. Figure 2(a-g) in the text should be changed to Fig. 3(a-g).

Author’s response: *We modified the figure captions in the paper.*

### Reviewer’s report 2: Marco Chierici

The authors state that “based on the clinical data, 259 patients were assigned in low risk group while 239 were assigned to the high risk group”: Unfortunately, this is not correct for two reasons. First, according to the provided clinical characteristics file, the high-risk patients are 176; secondly, the patients not marked as “high-risk” are not “low-risk” but can be either low or intermediate risk, thus they should be considered as “non high-risk”. Based on this classification, there are 13 patients among the non high-risk group that are not alive, differently from what stated in the paper. Please clarify this point and revise the results.

Author’s response: *We thank the reviewer’s thoughtful comment. In the original version of the paper, the 239 patients in the high-risk group was labeled based on our classification result from a companion paper using our algorithm. In this revision instead we focused on the 176 high-risk patients which are provided by clinical characteristics labeling from the CAMDA competition. And we recalculated the result showed substantial improvement over clinical staging. We have clarified this in the revision.*

About data preprocessing, were the microarray probes summarised at the gene level? If so, how? Parameter tuning in lmQCM was “based on previous work”, but this is unreferenced: Please provide a reference if available.

Author’s response: *We provided a reference to our previous paper in this revision.*

What about the rationale behind parameter tuning? Was it used in a similar condition? Was cross-validation used?

Author’s response: *Based on our extensive previous work, we have empirical knowledge about the range of four the parameters. We compared the different parameter in this range, lmQCM method used these parameters in the paper as they often led to balanced sizes of the gene modules with clear biological interpretations for individual modules.*

Regarding the parameter tuning in SNF: Did the authors try a grid search over the three SNF parameters, using cross-validation to evaluate the performance? How were the classification results evaluated in practice?

Author’s response: *We applied a grid search over the three SNF parameters.*

The references to figures in the main text are out of sync with the actual figure numbers, i.e. there are references up to Fig. 2 but there are 4 figures. Moreover, the caption for Fig. 3 is missing. Figure 5 A-d lacks a legend explaining the colors and is not referenced in the text; moreover, a different type of plot could better vehicle the information in a more compact way.

Author’s response: *We modified the figure captions.*

Please address minor typos such as missing spaces (as in the title of the methods section about SNF) and missing symbols (as the parameters in the SNF section). Some long sentences may be simplified (e.g., “To test the power of the combination (...) or the clinical stage information.” in conclusions).

Author’s response: *We corrected the typos and simplified long sentences.*

### Reviewer’s report 3: Dimitar Vassilev

Major merit of the study is the originality of the used methodology in the context of the applied procedures and approaches for emerging the dependance between the co-expressed genes and the potential of survival time prediction of the patients studied. All those methodological steps are composed in a workflow which has a potential capacity to be used in another cancer studies

Author’s response: *We thank the reviewer for the encouraging comments on this work.*

The suggested approaches for data integration based on mining gene co-expression network (GCN) is known and already applied in the studies, but the problem here is related to the selection of features in the context how to build and how to apply such a model (i.e. GCN) my remarks here can be related not to the applied method but again to the “tuning” of initial parameters and the potential of possible validation of them. And finally the method of similarity network fusion (SNF) for merging the eigengenes and to test their potential for functional biomarkers drops in semantics of the results in particular to the poorly explained functional annotation through the gene ontology enrichment. As it was presented and described, the workflow demands some clarification in terms of functionality of each step in it as well the total idea for validation of the functionality of the prognosticated biomarkers concerning the risk assessment for the survival time of the studied patients

Author’s response: *We provided more clarification for the functionality of each step in the workflow.*

There are also some potential remarks in using “our recently developed wighted network mining algorithm” based on local maximum click optimisation - where is not so clear for the point of view of defining of some initial parameters and their comparability

Author’s response: *Based on our extensive previous work, we have empirical knowledge about the range of for the parameters. We compared the different parameter in this range, lmQCM method used these parameters in the paper as they often led to balanced sizes of the gene modules with clear biological interpretations for individual modules.*

The submitted material needs of a thorough revision in English - both grammar and morphology which will improve significantly the and semantics of sentences. The illustrations are possibly the most questionable part of the study. I think the authors can renew the design of some of the figures which can be related in quite better manner to the obtained results (Fig. 5a, d)

Author’s response: *We checked the grammar and layout of the paper. Since* Fig. 5 *was confusing to readers, it was removed in the new version of the paper.*

The number and inclusion of references are limited and not enough for such an original work

Author’s response: *We added more references to support our work.*

Conclusions are as well recommended to be corrected in the context of the suggested workflow and the completeness of the work provided by that workflow

Author’s response: *We revised the description.*

Also avoiding for example such freely hanging phrases having obvious lack of comparability as “...which not only help achieve a more accurate survival prognosis...” will give the work better merit

Author’s response: *We revised the text accordingly.*

There are some obvious errors in grammar - in particular in the use of complex sentences and verbs with different tenses. The style can be improved also as a result of correction of the text in the context of spelling and grammar.

Author’s response: *We checked the spelling and grammar and made revisions accordingly.*

The level of the submitted material will be improved significantly by renewing some of the graphics (Fig. 5a, d)

Author’s response: *Since* Fig. 5 *was confusing to readers and was redundant to* Fig. 2*, it was removed in the new version of the paper.*

The data preprocessing and subsequent clusterization: Due to the highly unbalanced nature of the data there might be problems in defining categories as high or low risk. How the authors overcome the unbalancedness and the heterogenity of the data? Do the authors measure in someway the possible errors due to this problem?

Author’s response: *We thank the reviewer to point out the unbalanced data problem. If the reviewer refers to the clinical stage and clinical risk. Yes, there is unbalance issue. The number of patients labeled as stage 4 s and high risk are smaller/higher? (check it to be specific). However, we want to find survival-associated features. After we combined the deceased patients, the 105 patients deceased among total of 498 patients (21%), and among them, 92 patients are clinical high-risk in total of 176 clinical high-risk patients (55%). We think the sample sizes and proportions are appropriate for our statistical analysis. Furthermore, we used Regularized Cox proportional hazards model to calculate the risk indices of all patients. The median of risk indices of the training examples was used as a threshold to split patients into low-risk and high-risk groups. The same threshold was applied to classify the single held-out patient into one of the two groups, which means we were not using the same clinical categories as originally curated, which does not incur the unbalanced data issue. At last, we tested if these two groups have distinct survival outcome using Kaplan-Meier estimator and log-rank test. We divided patients into two groups (low and high group) where the median of each feature was used as a cut-off point. By using median as cutoff in the above two steps, we mitigated the unbalanced data issue in our survival association analysis.*

The suggested lmQCM approach for the purposes of defining GCN modules is interesting and having in mind some previous publications of the authors - it is a well tested method. However in the submitted material will be worth to explain what are exactly in this study the suggested four parameters Lambda, Alfa, t , and Beta. Definitely the fine tuning of these parameters can influence the final result in a large scale - it will be good to have authors explanation for these problems.

Author’s response: *Yes, as the reviewers pointed out, lmQCM has been applied to various types of cancer studies previously, and the meanings of the parameters were discussed in details in the previous publications* [10, 16]*. To further explain them, we added the following section to the manuscript: There are four parameters for lmQCM: γ, λ, t, and β. Among them, γ controls the threshold for the initiation of each new module, λ and t define the adaptive threshold of the module density to ensure proper stopping criterion for the greedy search for each module, and β is the threshold for overlapping ratio for merging. We used the same settings for our GCN module mining as in* [16] *for those parameters, which have been proved to generate functionally meaningful modules from multiple cancer datasets.*

The used Lasso-Cox model is a reasonable approach for defining the so-called risk index of the patients as it is given in the submitted material. The problem with such models as lasso regression (also elastic regression) can arose when they are applied to multivariate space parameters. Although the reduced parameter space by the eigengenes give some relaxation of such models it will be worth to explain the options how to control the Lasso-Cox risk index estimates from certain bias and what is the best way to validate this process?

Author’s response: *We thank the reviewers to point out this. To address the problem of applying lasso regression to multivariate space, we used a two-level cross validation (CV) strategy. The first level was leave-one-out CV. Namely, a single patient was chosen as test set, with the rest as training set. Then in the training set, we performed 10-fold CV to select the best regularization parameter. Regularized Cox proportional hazards model was built on the training set using the selected parameter, and based on the model, risk indices of all patients were calculated.*

The data preprocessing and subsequent clusterization: The Gene Ontology enrichment analysis might be not the major objective of the study but it is presented in a very limited manner. Using only a single tool for enrichment from an external knowledge source provokes a lot of questions about the accuracy of the defining (co)-expressed genes and in particular the accuracy of their annotation. My suggestion is that such an ontology enrichment can be extended at least to the major knowledge sources as Gene Ontology, NCBI, other. This can open some parallel to the study problems but from other view angle can extend and enrich all the suggested workflow by the authors.

Author’s response: *The online gene list enrichment tool ToppGene (*http://toppgene.cchmc.org*) developed by Cincinnati Children’s Hospital Medical Center* [20] *was used for all of the module functional enrichment analysis. ToppGene not only carries out enrichment analysis on standard Gene Ontology, it also generates enrichment results from more than 20 different sources including pathway databases, human and mouse phenotypes, NCBI PubMed, transcription factor binding sites, and drug information. We clarified in the revision.*

The last two part of the results section “Survival-associated feature selection using lasso-regularized Cox proportional hazard model” and the next one “Prognostic prediction based on integrative analysis” are written mostly as material and methods part. There are some problems again how are selected the features for Lasso-Cox model. The selection and subsequent clusterization of the selected eigengenes for obtaining some confidential biomarkers possibly needs some more methodological work. Nevertheless it would be good to get some explanation by the authors about the methodological solution and the obtained results more clearly: why it was done in this way?

Author’s response: *We thank the reviewer’s comment, it helps for us to rethink and better elucidate our purpose of study. To address this, we moved part of contents of the Results section “Survival-associated feature selection using lasso-regularized Cox proportional hazard model” and the “Prognostic prediction based on integrative analysis” to the Materials and methods section. We also added the details of our method and written in a more methodological form to explain our workflow.*

## Additional files

Additional file 1: **Table S1.** The table shown Gene symbols in unique co-expression modules, a comparison was performed between each pair of modules from microarray and RNA-seq. 17 GCN modules from microarray and 10 from RNA-seq are unique to its own data type. (XLS 36 kb)

Additional file 2: **Table S2.** Enrichment analysis for unique co-expression modules which are enriched with different biological processes, molecular functions, or specific pathways related to cancer physiology or to neurological functions (XLS 39 kb)

Additional file 3: **Figure S1.** The concordance index of the co-expression 17 unique microarray modules obtained from the training set in three conditions: the training set, testing set, and randomly selected gene modules with equal number of genes in testing set. (ZIP 75 kb)

Additional file 4: **Figure S2.** The concordance index of the co-expression 24 unique RNA-seq modules obtained from the training set in three conditions: the training set, testing set, and randomly selected gene modules with equal number of genes in testing set. (ZIP 101 kb)

## Abbreviations

GCN: Gene Co-expression Network
HR: High-risk
lasso-Cox: lasso-regularized Cox proportional hazards
lmQCM: local maximum Quasi-Clique Merging
NB: Neuroblastoma
SCC: Spearman Correlation Coefficient
SNF: Similarity Network Fusion
