# Supplementary material for: Integrative analysis based on survival associated co-expression gene modules for predicting Neuroblastoma patients’ survival time
Source: Biol Direct. 2019 Feb 13;14:4. doi: 10.1186/s13062-018-0229-2 (PMC6375203; doi:10.1186/s13062-018-0229-2)
Supplement: Supplementary file 3 — Figure S1. The concordance index of the co-expression 17 unique microarray modules obtained from the training set in three conditions: the training set, testing set, and randomly selected gene modules with equal number of genes in testing set. (ZIP 75 kb) [file 13062_2018_229_MOESM3_ESM.zip › Supplement R1R2.doc]

*Figure S1. The concordance index of the co-expression 17 unique microarray modules obtained from the training set in three conditions: the training set, testing set, and randomly selected gene modules with equal number of genes in testing set.*
