# Supplementary material for: Integrative analysis based on survival associated co-expression gene modules for predicting Neuroblastoma patients’ survival time
Source: Biol Direct. 2019 Feb 13;14:4. doi: 10.1186/s13062-018-0229-2 (PMC6375203; doi:10.1186/s13062-018-0229-2)
Supplement: Supplementary file 4 — Figure S2. The concordance index of the co-expression 24 unique RNA-seq modules obtained from the training set in three conditions: the training set, testing set, and randomly selected gene modules with equal number of genes in testing set. (ZIP 101 kb) [file 13062_2018_229_MOESM4_ESM.zip › Supplement R2.doc]

*Figure S2. The concordance index of the co-expression 24 unique RNA-seq modules obtained from the training set in three conditions: the training set, testing set, and randomly selected gene modules with equal number of genes in testing set.*
